# Linking Geological and Health Sciences to Assess Childhood Lead Poisoning from Artisanal Gold Mining in Nigeria

**DOI:** 10.1289/ehp.1206051

**Published:** 2013-03-22

**Authors:** Geoffrey S. Plumlee, James T. Durant, Suzette A. Morman, Antonio Neri, Ruth E. Wolf, Carrie A. Dooyema, Philip L. Hageman, Heather A. Lowers, Gregory L. Fernette, Gregory P. Meeker, William M. Benzel, Rhonda L. Driscoll, Cyrus J. Berry, James G. Crock, Harland L. Goldstein, Monique Adams, Casey L. Bartrem, Simba Tirima, Behrooz Behbod, Ian von Lindern, Mary Jean Brown

**Affiliations:** 1 U.S. Geological Survey, Denver, Colorado, USA; 2 Centers for Disease Control and Prevention, Atlanta, Georgia, USA; 3 Epidemic Intelligence Service, Centers for Disease Control and Prevention, Atlanta, Georgia, USA; 4 TerraGraphics Environmental Engineering, Moscow, Idaho, USA; 5 University of Idaho, Environmental Sciences Program, Moscow, Idaho, USA

**Keywords:** artisanal mining, environmental health, lead poisoning, mercury contamination, ore deposit geology

## Abstract

Background: In 2010, Médecins Sans Frontières discovered a lead poisoning outbreak linked to artisanal gold processing in northwestern Nigeria. The outbreak has killed approximately 400 young children and affected thousands more.

Objectives: Our aim was to undertake an interdisciplinary geological- and health-science assessment to clarify lead sources and exposure pathways, identify additional toxicants of concern and populations at risk, and examine potential for similar lead poisoning globally.

Methods: We applied diverse analytical methods to ore samples, soil and sweep samples from villages and family compounds, and plant foodstuff samples.

Results: Natural weathering of lead-rich gold ores before mining formed abundant, highly gastric-bioaccessible lead carbonates. The same fingerprint of lead minerals found in all sample types confirms that ore processing caused extreme contamination, with up to 185,000 ppm lead in soils/sweep samples and up to 145 ppm lead in plant foodstuffs. Incidental ingestion of soils via hand-to-mouth transmission and of dusts cleared from the respiratory tract is the dominant exposure pathway. Consumption of water and foodstuffs contaminated by the processing is likely lesser, but these are still significant exposure pathways. Although young children suffered the most immediate and severe consequences, results indicate that older children, adult workers, pregnant women, and breastfed infants are also at risk for lead poisoning. Mercury, arsenic, manganese, antimony, and crystalline silica exposures pose additional health threats.

Conclusions: Results inform ongoing efforts in Nigeria to assess lead contamination and poisoning, treat victims, mitigate exposures, and remediate contamination. Ore deposit geology, pre-mining weathering, and burgeoning artisanal mining may combine to cause similar lead poisoning disasters elsewhere globally.

In spring 2010, Médecins Sans Frontières (MSF) and Nigerian health officials conducting meningitis surveillance in Zamfara State, northwestern Nigeria, recognized an unprecedented outcome of artisanal (subsistence) gold extraction—a deadly outbreak of acute childhood lead poisoning (MSF 2012). They surmised that the outbreak resulted from artisanal processing of lead-rich gold ores, which had recently expanded in scope and become increasingly mechanized through use of gasoline engine-powered flourmills to grind the ores [MSF 2012; United Nations Environment Programme/Office for the Coordination of Humanitarian Affairs (UNEP/OCHA) 2010; von Lindern et al. 2011]. Representatives from MSF, Nigeria federal and state public health agencies, U.S. Centers for Disease Control and Prevention (CDC), TerraGraphics Environmental Engineering (TG), and the World Health Organization (WHO) determined that the outbreak has killed approximately 400 children < 5 years old and affected thousands more people, including > 2,000 children left with permanent disabilities (Dooyema et al. 2012; Lo et al. 2012; von Lindern et al. 2011).

In May 2010, at the request of the Nigerian Government through the U.S. Embassy in Abuja, a CDC emergency response team visited with TG two heavily affected villages, Dareta and Yargalma, to assess and help treat lead poisoning, characterize sources and routes of exposure to lead and other toxicants, and mitigate lead exposures (Dooyema et al. 2012). Using handheld X-ray fluorescence spectrometers (XRF) (Innov-XSystems, Woburn, MA, USA; and Thermo-Scientific Niton, Billerica, MA, USA) [U.S. Environmental Protection Agency (EPA) 2007], Dooyema et al. (2012) measured extreme concentrations of soil lead (often > 100,000 ppm) and soil mercury (up to 4,600 ppm). They found that surviving children < 5 years old had blood lead levels (BLL) up to 370 µg/dL—extraordinary levels given that the CDC recommends BLL be < 5 µg/dL (CDC 2012). Dooyema et al. (2012) also found elevated blood manganese levels up to 41 µg/L, with 66% of samples above their cited 7.7–12.1 µg/L reference range. In October–November 2010, CDC and TG field teams assessed 74 additional Zamfara villages (Lo et al. 2012; von Lindern et al. 2011), finding evidence of ore processing and/or lead contamination in more than half the villages and identifying 1,500–2,000 additional children < 5 years old as lead poisoned and in need of treatment.

Observations made by and photographs taken by the field teams indicated there were opportunities for exposures to lead and other contaminants in all stages of mining and processing [MSF 2012; von Lindern et al. 2011; see also Supplemental Material, Figure S1A–C (http://dx.doi.org/10.1289/ehp.1206051)]. Quartz (crystalline silica)–rich veins in bedrock (Garba 2003) were mined by hand from near-surface workings. At the mines, ores were sorted into “gold” and “lead” ores based on visual absence or presence of shiny gray lead sulfides. “Gold” ores were transported in cloth bags to villages, where families purchased them for processing. Initial processing involved breaking down the ore using hammers. Broken ore fragments were then ground using mortars and pestles or gasoline-powered flourmills, which were also used to process grain, spice, and herb foodstuffs when not used for ore grinding. The mechanized grinding generated large amounts of dust. Ground ores were then sluiced and washed near village water sources to concentrate gold particles. These concentrates were amalgamated with liquid mercury by hand in cooking pots. Waste waters and solids produced by sluicing and amalgamation were either disposed of nearby or reprocessed. The gold–mercury amalgam was smelted in open fires to volatilize the mercury. Some ore processing and storage of ores in porous cloth bags occurred in family compounds near where children ate, played, and slept. Younger children were often present during processing, and older children worked at the processing. Soils contaminated by sluicing and washing wastes were used to make adobe bricks for building construction.

The CDC/TG field teams collected an extensive suite of raw (unprocessed) ore samples, ore samples from various processing steps, composite soil samples within and outside villages, and “sweep” samples of dust and loose soils from dirt floors in family compounds near where children ate or slept (Dooyema et al. 2012). TG obtained samples of raw and processed grain, spice, and medicinal herb foodstuffs from residents’ household supplies or from local public markets.

At CDC’s request, the U.S. Geological Survey (USGS) has collaborated with CDC and TG to carry out an interdisciplinary earth and health science analysis of the samples, with a focus primarily on those collected in Dareta and Yargalma. The purpose of this study is to summarize results and implications of these analyses.

## Methods

Methods by which the different sample types were collected in Zamfara are described in Supplemental Material, Field Sampling Methods (http://dx.doi.org/10.1289/ehp.1206051).

We analyzed the samples at USGS laboratories in Denver, Colorado (USA), incorporating appropriate quality assurance/quality control analyses of standard reference materials, duplicate sample splits, analytical duplicates, and blanks. See Supplemental Material, Table S1, for analytical method details and references (http://dx.doi.org/10.1289/ehp.1206051).

Nearly 200 spot chemical analyses were performed in the laboratory by handheld XRF on > 50 raw ore samples, to assess natural chemical heterogeneities within and between the samples.

Representative splits of all processed ores, soils, and sweep samples were analyzed for multiple parameters. Quantitative particle size distribution of samples sieved to < 2 mm was measured by laser diffraction. Powder X-ray diffraction was used to qualitatively identify relative weight proportions of specific minerals present above the detection limit of approximately 2 weight %. Total chemical concentrations of 42 elements were analyzed using inductively coupled plasma–mass spectrometry (ICP-MS). Total mercury was analyzed using continuous flow–cold vapor–atomic fluorescence spectroscopy (CVAFS).

Scanning electron microscopy (SEM) was performed on a subset of raw ores, processed ores, soils, sweep samples, and grain samples to determine individual particle mineralogy, chemistry, size, and shape.

Deionized water extractions were performed on a subset of processed ores, soils, and sweep samples to model constituent release into surface waters (Hageman 2007).

*In vitro* bioaccessibility assessments (IVBA) were performed on a subset of processed ores, soils, and sweep samples to model toxicant bioaccessibility and bioavailability along ingestion exposure pathways (Drexler and Brattin 2007; Morman et al. 2009). Bioaccessibility measures the amount of a toxicant that is dissolved in the body’s fluids and is available for uptake into the body’s circulatory system, whereas bioavailability measures the amount of a toxicant that is absorbed by the body and transported to a site of toxic action [see references in Plumlee and Morman (2011)]. The IVBA we used leaches samples with simulated gastric fluid for 1 hr at 37°C [see Supplemental Material, Table S1 (http://dx.doi.org/10.1289/ehp.1206051)], and is based on the Drexler and Brattin (2007) method, validated for lead against juvenile swine uptake. The juvenile swine uptake model is a proxy for relative lead bioavailability in humans that integrates both lead dissolution in the stomach acids and uptake via the intestines (Casteel et al. 2006). This IVBA has not been validated against swine uptake for other toxicants such as arsenic, mercury, and manganese, but nonetheless provides useful insights into their potential gastric bioaccessibility (Plumlee and Morman, 2011).

Plant foodstuff samples were analyzed for 40 elements by inductively coupled plasma–atomic emission spectroscopy (ICP-AES) and mercury by CVAFS.

Analytical methods used for total and leachate chemical analyses provided concentration data for many potential elemental toxicants in addition to lead and mercury, such as arsenic, antimony, manganese, iron, aluminum, cadmium, copper, zinc, and nickel.

## Results

Unweathered (primary) vein ores were dominated by quartz (crystalline silica), with variable amounts of galena (lead sulfide) and minor amounts of pyrite (iron sulfide), chalcopyrite (copper–iron sulfide), and arsenopyrite (iron–arsenic sulfide) [see Supplemental Material, Figure S1D–G, Table S2 (http://dx.doi.org/10.1289/ehp.1206051)]. Natural weathering and oxidation of the vein ores over millennia before mining partially converted primary sulfide minerals into complex secondary mineral assemblages with abundant lead carbonates and lead phosphates (see Supplemental Material, Figure S1D–G, Table S2).

Dareta and Yargalma sweep and soil samples contained broken particles of the same complex suite of primary and secondary lead minerals as unprocessed and ground vein ores [see Supplemental Material, Table S2 (http://dx.doi.org/10.1289/ehp.1206051)]. This mineralogical fingerprint confirmed ore processing as the source for contamination.

**Figure 1:**
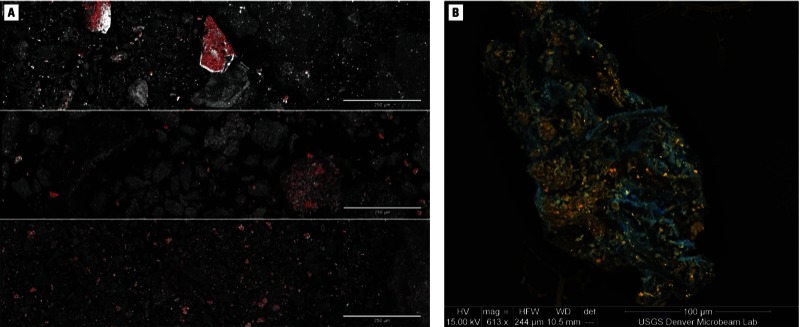
(*A*) Backscatter electron (BSE) scanning electron microscope (SEM) images of Nigeria ground ore (upper), eating area sweep (middle), and soil (lower) samples with overlain element maps for lead (in red). In all images, the brighter gray indicates higher mean atomic number. Bar = 250 µm. (*B*) BSE field emission SEM image of a cluster of plant fibers and mineral particles found in a grain sample (ground by a flour mill in Zamfara) having 3 ppm total lead. Elongated plant fibers are light to dark blue. Bright orange particles are lead carbonates, lead oxides, and lead phosphates. Pale orange/blue particles containing iron, chromium, and nickel are steel particles abraded from flourmill grinding plates. The cluster formed during grinding, with the fiber bundle attracting and trapping the mineral and metal fragments. Bar = 100 µm.

Quantitative particle size analysis and visual estimation by SEM element mapping (Figure 1A) show that > 90% of lead-rich particles in the ground ores, soil samples, and sweep samples were < 250 µm in diameter, regarded as a maximum size for incidental ingestion by hand–mouth transmission (Drexler and Brattin 2007). Visual estimation using SEM element mapping shows that > 50% of the lead-rich particles were also < 10–15 µm (Figure 1A), and could therefore be inhaled into at least the upper respiratory tract, where many would likely be trapped and cleared by mucociliary action.

**Figure 2:**
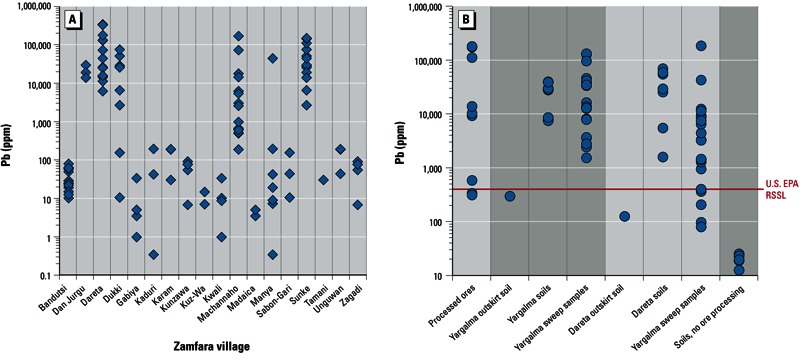
(*A*) Total lead (Pb) concentrations (measured in the laboratory using handheld XRF) in raw ore samples collected from different Zamfara villages. Multiple spot analyses were made on multiple ore samples from each village to account for substantial mineralogical heterogeneities within samples. (*B*) Total lead concentrations in processed ores, soils, and sweep samples from Dareta and Yargalma, as measured by ICP-M. Red line indicates U.S. EPA (2011a) RSSL for lead (400 ppm).

Laboratory handheld XRF spot analyses of raw ore samples collected from 18 villages indicated that the ores being processed varied considerably in their lead content within samples, and between different villages and mine sources (Figure 2A). ICP-MS analyses measured up to 180,000 ppm lead in processed ore samples from Dareta and Yargalma (Table 1, Figure 2B).

**Table 1:** Summary of USGS laboratory analytical results for total chemical composition.

Sample type (*n* samples)	Lead ppm [range (median)]	Mercury ppm [range (median)]	Manganese ppm [range (median)]	Arsenic ppm [range (median)]	Antimony ppm [range (median)]
Raw ores (189 spot analyses)	<0.3–333,000 (185)	0.5–25 (4.0)	<17–1,447 (16.7)	<0.5*–*73,497 (57.4)	<1–3,188 (62)
Processed ores
Crushed, ground, washed ores (6)	305–180,000 (10,200)	0.1–0.9 (0.3)	71–1,320 (722)	2.5–69 (14)	6.9–60 (22.5)
Sluiced ores (1)	112,000	7	550	110	366
Soils from ore sluicing, ore washing areas
Dareta (3)	5,420–58,900 (54,400)	2.1–12.6 (2.4)	694–859 (749)	8.5–140 (140)	31.2–435 (389)
Yargalma (5)	27,700–39,200 (29,800)	13.8–15.1 (14.5)	235–413 (391)	20.4–76 (22)	174–344 (279)
Sweep samples
Dareta (17)	78–185,000 (3,250)	0.05–68.1 (0.5)	256–1,040 (500)	1.5–27 (3.8)	0.7–79 (9.1)
Yargalma (15)	1,510–132,000 (33,700)	1.0–44.0 (5.9)	196–649 (309)	4.7–270 (44)	13.7–1,250 (189)
Village soils
Dareta (4)	1,560–69,700 (27,400)	0.7–15.2 (2.4)	703–1,060 (851)	5.3–150 (31.5)	9.5–431 (89)
Yargalma (3)	7,450–8,490 (7,630)	2.7–3.9 (2.9)	433–625 (470)	22–76 (33)	54–144 (77)
Village outskirt soils
Dareta (1)	122	0.3	620	3.3	431
Yargalma (1)	293	0.2	284	2.8	3
Village soils, no ore processing (5)	12–25 (19)	0.01–0.1 (0.02)	113–419 (255)	2–11.2 (2.4)	0.1–6.9 (0.3)
RSSL (U.S. EPA 2002a, 2011a)	400	10–23^*a*^	390	0.39	31
Zamfara plant foodstuff samples
Processed samples (16)	0.1–146 (1.5)	0.02–0.45 (0.07)	7.5–136 (22.3)	<0.05–0.91 (739)	0.7–1.4 (0.95)
Raw samples (23)	<0.05–1.86 (0.39)	0.01–0.15 (0.06)	3.4–242 (14.6)	<0.05*–*0.45 (0.27)	0.3–1.4 (0.94)
Plant standard reference materials
NIST wheat 1567a	<0.05	0.006	7.6	<0.05	1
NIST rice 1568a	<0.05	0.0005	18.5	<0.05	0.9
NIST samples from National Institute of Standards and Technology, Gaithersburg, MD, USA. Analytical methods used for raw ores: handheld XRF in lab; for processed ores, all soils, sweep samples: ICP-MS, mercury by CVAFS; for plant foodstuffs: ICP-AES, mercury by CVAFS. ^***a***^Range is for elemental (10) to salt (23) forms.					

ICP-MS analyses found that all Dareta and Yargalma soil samples and most sweep samples had extreme lead concentrations (up to 185,000 ppm), far above the U.S. EPA (2011a) residential soil screening level (RSSL) of 400 ppm (Table 1, Figure 2). In contrast, lead concentrations in background soils from five villages without gold processing were < 25 ppm. Composite soil samples collected on the outskirts of Dareta and Yargalma (~ 100 m from the edge of each village) had elevated lead concentrations (122 and 293 ppm, respectively) (Table 1), indicating that processing-related contamination extended beyond village limits. Soils used to make adobe bricks (from ore washing areas) had lead levels as high as 58,900 ppm.

Total lead concentrations measured with ICP-MS in soil and sweep samples with total lead > approximately 400 ppm were generally twice the concentrations measured on the same samples in the field by CDC/TG using handheld XRF [see Supplemental Material, Figure S2A (http://dx.doi.org/10.1289/ehp.1206051)]. TG has found such field underestimation to be common (von Lindern I, unpublished data), possibly resulting from sample compositing/sieving effects, summer heat impacts on the instruments, and/or lack of field XRF calibration standards having extreme lead concentrations. For the few samples analyzed with lead < approximately 400 ppm, lab ICP-MS results were variously greater than, close to, or less than the field XRF results (see Supplemental Material, Figure S2A inset).

Extreme total mercury concentrations measured in soil and sweep samples (up to 4,600 ppm measured in the field by XRF; up to 68.1 ppm by laboratory CVAFS) were higher than levels measured in raw and ground ores, and were well above the U.S. EPA elemental mercury RSSL of 10 ppm [Table 1; see also Supplemental Material, Figure S3A (http://dx.doi.org/10.1289/ehp.1206051)]. Hence, mercury contamination resulted predominantly from the amalgamation processing. Substantially greater concentrations of mercury were measured in soil and sweep samples by field XRF compared with those measured by CVAFS for the same samples (see Supplemental Material, Figure S2B), indicating that mercury was volatilized from the samples after sample collection.

Manganese concentrations in processed ores, background soils, village soils, and sweep samples (up to 1,320 ppm) commonly exceeded the U.S. EPA RSSL of 390 ppm (U.S. EPA 2011a), and were generally higher than concentrations measured in soils from villages without ore processing [Table 1; see also Supplemental Material, Figure S3B (http://dx.doi.org/10.1289/ehp.1206051)]. Concentrations of arsenic (up to 270 ppm) and antimony (up to 1,250 ppm) in some soil and sweep samples greatly exceeded U.S. EPA RSSLs of 0.39 (U.S. EPA 2011a) and 31 ppm (U.S. EPA 2002a), respectively (Table 1; see also Supplemental Material, Figure S3C–D). Concentrations of other potential environmental or human toxicants such as cadmium, zinc, copper, and nickel were well below U.S. EPA RSSLs.

Deionized water leach tests on soil and sweep samples produced moderately alkaline leachates with pH from 7.7 to 9.1. Metal toxicants were not appreciably water soluble, with a maximum of 0.018% of total lead, 0.7% of total mercury, 2.9% of total manganese, < 0.2% of total arsenic, and 0.03% of total antimony being solubilized by water leaching of any given sample (Table 2).

**Table 2:** Ranges in percentage of water-leachable elemental toxicants measured in different sample types.

Sample type (*n* samples)	Lead % leached	Mercury % leached (*n* RL)	Manganese % leached	Arsenic % leached (*n* RL)	Antimony % leached (*n* RL)
Processed ores (3)	0.00015–0.018	< 0.1–0.7 (1)	0.09–2.9	< 0.2 (3)	< 0.01 (3)
Washing area soil (1)	0.01	0.1	0.17	< 0.2 (1)	0.03
Sweep samples (3)	0.003–0.008	0.03–0.3	0.06–0.1	< 0.2 (3)	< 0.01–0.01 (2)
Percent leached = 100 × (ppm leached)/(ppm total in solid), where (ppm leached) = (mg/kg leachate) × (20 kg ­leachate/1 kg solid). (*n* RL) indicates number of samples with concentration below analytical method reporting limit.					

**Figure 3:**
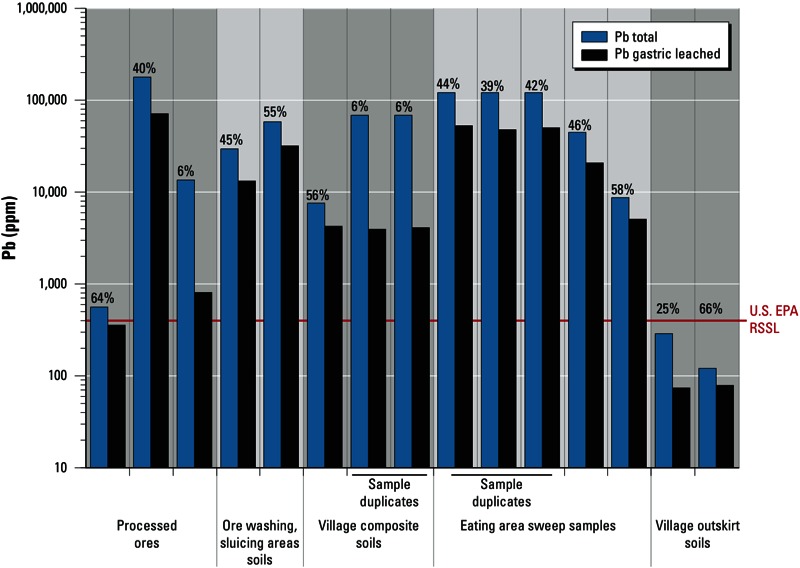
Total lead (Pb) concentrations (ppm mass basis; mg lead/kg solid) and simulated gastric fluid–leachable lead concentrations (ppm mass basis; calculated as (mg lead/kg leachate) × (100 kg leachate/1 kg solid)] in Zamfara samples. Each bar pair represents results for a single sample. Percentage of bioaccessible lead is listed above the paired bars for each sample, and was calculated by dividing the SGF-leachable concentration by the total concentration for the sample, and then multiplying by 100. Horizontal red line indicates U.S. EPA (2011a) RSSL for lead (400 ppm).

Lead was generally highly gastric bioaccessible, with 39–66% of the total lead solubilized in an hour from 9 of 12 samples analyzed (Table 3, Figure 3). The highest percent bioaccessibility was measured in a less heavily contaminated village outskirt soil. Manganese was also generally quite gastric bioaccessible, with 6–43% of the total manganese solubilized. However, mercury (< 0.9% of total), arsenic (< 2.1% of total), and antimony (< 1.4% of the total) were not appreciably gastric bioaccessible (Table 3).

**Table 3:** Ranges in percentage of gastric-bioaccessible elemental toxicants measured in different sample types.

Sample type (*n* samples)	Lead % bioaccessible	Mercury % bioaccessible	Manganese % bioaccessible	Arsenic % bioaccessible (*n *RL)	Antimony % bioaccessible (*n *RL)
Processed ores (3)	6–64	0.1–0.9	9.0–43	< 1–2.1 (2)	< 0.05 (3)
Washing, sluicing area soils (2)	45–55	0.3	15–23	< 1–1.9 (1)	0.3–1.4
Village composite soils (2)	6–56	Not analyzed	19–31	< 1 (2)	< 0.05 (2)
Sweep samples (3)	39–58	0.0009–0.2	11.0–41	< 1–1.8 (1)	< 0.05–0.2 (1)
Village outskirt soil (2)	26–66	Not analyzed	6.0–15	< 1 (2)	< 0.05 (2)
Percent bioaccessible = 100 × (ppm leached)/(ppm total in solid), where (ppm leached) = (mg/kg leachate) × (100 kg leachate/1 kg solid). (*n* RL) indicates number of samples with concentration below analytical detection limit.					

Chemical analyses of 39 rice, corn, spice, and medicinal herb samples found that all 16 processed samples and 19 of 23 raw samples were lead-contaminated (from 0.1 to 146 ppm) compared with plant standard reference materials [Table 1; see also Supplemental Material, Figure S3E (http://dx.doi.org/10.1289/ehp.1206051)]. The same suite of lead carbonates and other secondary lead minerals was found in the plant foodstuffs as in the ores, soils, and sweep samples (Figure 1B). This mineralogical fingerprint confirms that stored foodstuffs were contaminated by ore-processing dusts and that grains were contaminated when ground using flourmills also used for ore grinding. Elevated concentrations of mercury from 0.01 to 0.45 ppm (Table 1; see also Supplemental Material, Figure S3F) found in 10 of 16 processed and 8 of 23 raw foodstuff samples also indicate processing-related contamination, possibly from airborne mercury, use of flourmills for both food and ore regrinding, and foodstuff storage in cooking pots used for amalgamation.

## Discussion

Our results document that ore deposit geology and mechanized ore grinding were fundamental causes of this unusual lead poisoning outbreak linked to artisanal gold mining. Not only can the vein gold ores be relatively lead rich, but much of the lead occurs in minerals with enhanced gastric bioaccessibility caused by natural weathering of the ores over millennia before mining. Weathering transformed minimally gastric-bioaccessible primary lead sulfides into abundant, highly gastric-bioaccessible secondary lead carbonates and moderately gastric-bioaccessible lead phosphates (Casteel et al. 2006). Mechanized ore grinding greatly increased both the volumes of ore that could be processed and the amounts of lead-rich particles having optimal size for dispersion as dusts and particle uptake by hand–mouth transmission or inhalation. By creating many particles < 10–15 µm in size, grinding also greatly enhanced the surface area per mass of ingested particles, thereby enhancing dissolution rates [see references in Plumlee and Morman (2011)].

*Lead exposure pathways*. Data are lacking to do a Zamfara-specific integrated exposure uptake biokinetic model for lead in children (U.S. EPA 2002b) because the model uses a series of U.S.-centric assumptions on dietary intake, living in houses with nonsoil floors, and other factors. However, our results can be used to help infer relative importance of various lead exposure pathways.

**Figure 4:**
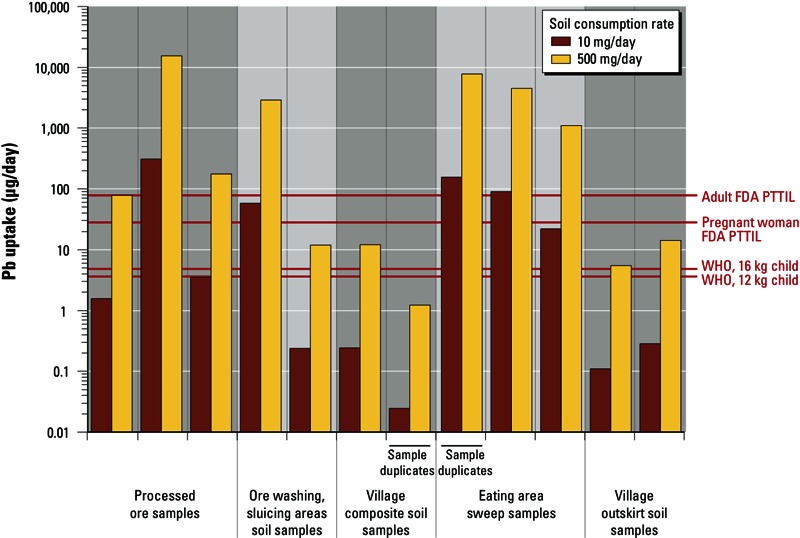
Calculated daily lead uptake assuming exposures to processed ores, soils, and sweep samples from Zamfara. For each sample, measured gastric bioaccessibility of lead (from Figure 3) was translated into gastric bioavailability using equations in Drexler and Brattin (2007) [see Supplemental Material, Lead Uptake Calculations (http://dx.doi.org/10.1289/ehp.1206051)]. The gastric bioavailability was then translated into daily uptake amount using soil consumption rates for young children from the literature (U.S. EPA 2011b). Brown bars assume 10-mg/day soil consumption (unrealistically clean conditions), and yellow bars assume 500-mg/day soil consumption (very dusty but plausible conditions). Bar pairs show results for the corresponding samples in Figure 3, except that bar pairs labeled as sample duplicates are averages of sample duplicate analyses. Horizontal red lines show WHO (2011) dietary exposure levels for 12-kg child (3.6 µg/day) and 16-kg child (4.8 µg/day) known to adversely affect health, and FDA PTTILs (FDA 1993) for pregnant or lactating women (25 µg/day) and adults (75 µg/day). Although called “intake levels,” PTTILs are in effect uptake levels because they were derived assuming 48% absorption.

Figure 4 shows results of calculations estimating plausible ranges in daily lead uptake from inadvertent ingestion of the different processed ore, soil, and sweep samples we analyzed.

We calculated lead uptake levels using soil consumption rates, our bioaccessibility results (Figure 3), and the method described by Drexler and Brattin (2007) to convert bioaccessible lead into bioavailable lead for uptake modeling. We used published soil consumption rates (e.g., U.S. EPA 2011b) of 10 and 500 mg/day—a range for children under clean (unlikely for the villages) to extremely dusty (more plausible) conditions. See Supplemental Material, Lead Uptake Calculations for details (http://dx.doi.org/10.1289/ehp.1206051).

The results (Figure 4) suggest that inadvertent ingestion could plausibly result in lead uptake as high as several tens to several thousands of micrograms per day, depending upon time spent by exposed persons in contaminated eating areas or ore processing areas. These lead uptake levels can vastly exceed the dietary lead exposure levels (0.3 µg/kg body weight/day) that WHO (2011b) recognizes as causing adverse health impacts in young children. They can also substantially exceed U.S. Food and Drug Administration (FDA) provisional total tolerable intake levels (PTTILs) (FDA 1993) for pregnant or lactating women (25 µg/day) and adults (75 µg/day). Potentially significant lead uptake could even occur from less heavily contaminated soils with total lead concentrations below the U.S. EPA 400-ppm RSSL. This is demonstrated by the village outskirt soil sample having 120 ppm total lead with 66% gastric bioaccessibility, which under plausibly high soil consumption rates could cause problematic lead uptake (Figures 3 and 4).

Additional lead uptake not accounted for by ingestion via hand–mouth transmission would also occur via ingestion of inhaled lead particles that are cleared by mucociliary action from the respiratory tract and swallowed (Plumlee and Morman 2011).

Dooyema et al. (2012) and UNEP/OCHA (2010) found evidence of processing-related contamination in samples of potable well waters and surface waters from the villages studied, with many having lead concentrations above the WHO (2011a) guideline of 10 µg/L. Most contaminated well water samples had 10–20 µg/L dissolved lead, several had up to several hundred micrograms per liter dissolved lead, and two (Dooyema et al. 2012) had total lead levels of 520 and 1,500 µg/L. Low levels of water-soluble lead found in ores and soils by our water leach tests suggest the highest lead concentrations in water likely resulted from suspended particles such as lead carbonates. Three-year-olds drinking 1.3 L of water a day (U.S. EPA 2011b) from most sampled wells could consume from 10 to several hundred micrograms of lead per day, with locally higher consumption rates of up to 2,000 µg/day for water from the most contaminated wells. Our results substantiate UNEP/OCHA (2010) conclusions that consumption of lead-contaminated water, although substantial, is a subordinate exposure pathway to incidental ingestion of lead-contaminated soils or dusts.

Consumption of plant foodstuffs contaminated by lead particles from the processing is plausibly a lesser but still measureable contribution to total lead uptake. Other exposure pathways that still need evaluation include consumption of garden vegetables grown in contaminated soils; consumption of milk or meat from cows, goats, and chickens that forage in contaminated areas; consumption of breast milk from mothers exposed to contamination; and exposures to particles abraded from adobe bricks made with lead-contaminated wastes.

*Additional health concerns*. Deaths of and adverse health impacts on children < 5 years old led responding organizations to focus on preventing child death and illness from lead poisoning. However, our results indicate that older children and adults who process ores, pregnant women and their unborn children, and breastfed infants are also at risk for lead poisoning.

The potential environmental and health effects of mercury contamination from amalgamation processing should be further assessed in Zamfara, including environmental conversion of inorganic mercury to more toxic methylmercury; dermal mercury exposures during amalgamation; mercury vapor inhalation during amalgam smelting; and mercury uptake from contaminated food.

Elevated blood manganese levels may also be a health concern. Results indicate that manganese uptake from incidental ingestion of soils and dusts contaminated by ore processing is a plausible exposure route. Uptake of bioaccessible manganese, mercury, arsenic, and antimony from inhaled dusts in the sinuses, upper respiratory tract, and lungs is also plausible and could be evaluated with IVBAs using lung fluid simulants (Plumlee and Morman 2011). Contamination of local wetlands, ponds, and rivers by dusts and sluicing wastes could be a pathway for all toxicants into the aquatic food chain.

Because the ores are dominated by crystalline silica, silicosis and related diseases (e.g., silicotuberculosis) could become long-term health problems in ore processors who do not use appropriate respiratory protection or dust control measures (National Institute for Occupational Safety and Health 2002). Children and other bystanders to the processing may also be at risk.

Nascent research indicates that multiple-toxicant exposures can either exacerbate or counteract health effects of individual toxicants [Agency for Toxic Substances and Disease Registry (ATSDR) 2004]. No toxicological profile exists for the mix of all toxicants identified in this study. However, synergistic toxicological effects on neurodevelopment in early childhood have been found following lead and manganese coexposures (Henn et al. 2012). Other synergistic interactions such as lead–arsenic and lead–methylmercury have also been noted (ATSDR 2004).

*Aiding the crisis response in Zamfara*. MSF, Blacksmith Institute, TG, CDC, UNICEF, and Nigerian government agencies have implemented advocacy, education, remediation, and risk mitigation strategies in Dareta, Yargalma, and five other villages (MSF 2012; von Lindern et al. 2011). These include working with the local emirate to move gold processing out of family compounds and away from village centers; removing the top several centimeters of contaminated soil and replacing with clean soil; providing chelation therapy for > 2,000 severely affected children in remediated villages; and educating villagers on safe ore processing practices. Because of logistical challenges faced by field teams, the number of contaminated villages requiring remediation may be even greater than indicated by the 2010 screening survey of 74 Zamfara villages (Lo et al. 2012). Insights from our study help inform and refine these efforts.

A systematic geological assessment of gold mines throughout the region is needed to screen lead-poor deposits from lead-rich deposits (Figure 2A) and identify deposits with abundant lead carbonates. Artisanal mining and processing could ideally focus on lead-poor ores. However, economic considerations will likely drive processing of all gold-bearing ores regardless of lead content. Hence, methods are needed to identify ores that require mitigation of lead contamination and exposures during mining and processing.

Unfortunately, lead carbonates and lead oxides are not readily identifiable by eye. A chemical spot detection test (Esswein and Ashley 2003) successfully identifies lead-rich samples from the area [see Supplemental Material, Figure S4 (http://dx.doi.org/10.1289/ehp.1206051)], and could help workers identify lead-rich ores that lack visually distinctive lead sulfides.

Lack of laboratory facilities and need for rapid decisions in remote areas make handheld field XRF an essential field screening tool. It has helped identify dozens of Zamfara villages with lead contamination (Lo et al. 2012), and is key to guide remediation decisions and assess remediation effectiveness (von Lindern et al. 2011). Based on prior experience, TG field crews knew that field XRF underestimates lead concentrations (von Lindern I, unpublished data), and factored this into assessment or remediation decisions made based on field XRF results. Our results comparing field XRF and ICP-MS values for lead across wide concentration ranges will help users better understand the accuracy of XRF when making field decisions. Probable mercury loss from samples following sampling indicates that field XRF is the best way to assess mercury contamination.

The elevated levels of highly bioaccessible lead found in village outskirts soils compared to those in soils not affected by ore processing (Figures 2B, 3) indicates that XRF testing for contamination should be extended to > 100 m outside villages. Less heavily contaminated soils with lead concentrations < 400 ppm may result in problematic lead uptake under dusty conditions.

Continued education of villagers and workers is needed to help ensure that soils contaminated by processing wastes are not used to make adobe bricks; mortars/pestles, flourmills, and cooking pots are not used for ore processing, food processing, and cooking; contaminated ore storage sacks are not reused for food storage or bedding; and stored foods are protected from processing-related contamination. Education on removal of particulate lead from potable well waters by allowing suspended sediments to settle before consumption should help lessen lead intake via water consumption.

Relief organizations have suggested alternative gold extraction methods to reduce lead and mercury contamination, including wet processing to minimize dust generation, retorts to reduce mercury vapor emission during amalgam smelting, and cyanide-based chemical extraction. These alternatives have benefits but could inadvertently cause new waste disposal issues, contamination sources, and exposure pathways. For example, cyanide extraction requires ore breaking (with accompanying dust generation), and if done improperly could contaminate local waters with dissolved cyanide, lead, and arsenic. Because sulfides in the ores reduce cyanide extraction efficiency, workers may resort to ore roasting pretreatment, which would cause widespread contamination by deleterious sulfur dioxide gas and airborne roaster particulates with highly bioaccessible lead (Plumlee and Morman 2011).

*Global health implications*. Price increases in gold and other metals have caused artisanal mining to burgeon globally, increasing the potential for lead poisoning outbreaks beyond Nigeria. For example, tens of thousands of people have been affected by lead poisoning at Kabwe, Zambia, which resulted from artisanal re-mining of and exposures to wastes from historical lead–zinc mining and smelting (Branan 2008). By understanding ore deposit geology and climate controls on pre-mining ore weathering (Plumlee and Morman 2011), geologists can help identify other artisanal mining areas that may be at higher risk for lead poisoning and need medical surveillance.

Of highest risk are lead-bearing gold deposits and lead–zinc deposits that either contain abundant carbonate minerals (as at Kabwe) or are located in dry climates where surface and ground waters are relatively alkaline (as in Zamfara). In these situations, highly bioaccessible secondary lead carbonates are likely to be abundant. In contrast, some other gold deposit types mined artisanally are lead poor and pose low lead poisoning risk. However, they may contain high levels of arsenic or other toxicants that are of potential health concern (e.g., Ashanti gold belt, Ghana) (Hilson 2002). Artisanal re-mining in historical mining camps with prior uncontrolled smelting or roasting of lead-bearing ores (e.g., Kabwe) will have high bioaccessible lead and high lead poisoning risk regardless of deposit type (Plumlee and Morman, 2011).

## Conclusions

The results of the present study support the conclusion that the fatal lead poisoning outbreak in northern Nigeria resulted from contamination of soils, living areas, water supplies, and foodstuffs by the processing of weathered, lead-rich gold ores containing abundant, highly gastric-bioaccessible secondary lead carbonate minerals. The dominant exposure pathway is incidental ingestion of lead-rich soil and dust particles by hand–mouth transmission and of inhaled dust particles cleared from the respiratory tract. Lesser but still significant pathways (each of which alone would be problematic) include consumption of water and foodstuffs contaminated by the processing. Although acute lead poisoning of young children has been the most immediate and severe consequence, older children, adult workers, pregnant women and their unborn children, and breastfeeding infants are also at risk. Other contaminants (manganese, arsenic, antimony, crystalline silica) may pose additional health threats. Lead poisoning may occur elsewhere in the world from artisanal mining in geologically and climatically favorable areas.

This study underscores the value of collaborative interdisciplinary studies involving health, geological, and engineering scientists. This scientific input will aid development of evidence-based policies on artisanal resource extraction that greatly reduce environmental contamination and adverse health impacts.

## Supplemental Material

(6.3 MB) PDF

## References

[r1] ATSDR (Agency for Toxic Substances and Disease Registry). (2004). Guidance Manual for the Assessment of Joint Toxic Action of Chemical Mixtures.. http://www.atsdr.cdc.gov/interactionprofiles/IP-ga/ipga.pdf.

[r2] Branan N. (2008). Mining Leaves Nasty Legacy in Zambia.. http://www.geotimes.org/jan08/article.html?id=nn_zambia.html#top.

[r3] Casteel SW, Weis CP, Henningsen GM, Brattin WJ (2006). Estimation of relative bioavailability of lead in soil and soil-like materials using young swine.. Environ Health Perspect.

[r4] CDC (Centers for Disease Control and Prevention). (2012). Response to the Advisory Committee on Childhood Lead Poisoning Prevention Report, Low Level Lead Exposure Harms Children: A Renewed Call for Primary Prevention. Morb Mortal Wkly Rep MMWR 61:383.. http://www.cdc.gov/mmwr/preview/mmwrhtml/mm6120a6.htm.

[r5] Dooyema C, Neri A, Lo Y-C, Durant J, Dargan PI, Swarthout T (2012). Outbreak of fatal childhood lead poisoning related to artisanal gold mining in northwestern Nigeria, 2010.. Environ Health Perspect.

[r6] Drexler JW, Brattin WJ (2007). An *in vitro* procedure for estimation of lead relative bioavailability, with validation.. Human Ecol Risk Assess.

[r7] Esswein EJ, Ashley K. (2003). Lead in Dust Wipes by Chemical Spot Test, NIOSH Method 9105. In: NIOSH Manual of Analytical Methods, 4th ed.. http://www.cdc.gov/niosh/docs/2003-154/pdfs/9105.pdf.

[r8] FDA (U.S. Food and Drug Administration) (1993). Lead-soldered food cans.. Fed Reg.

[r9] Garba I (2003). Geochemical characteristics of mesothermal gold mineralisation in the Pan-African (600 ± 150 Ma) basement of Nigeria.. Appl Earth Sci.

[r10] Hageman PL. (2007). U.S. Geological Survey field leach test for assessing water reactivity and leaching potential of mine wastes, soils, and other geologic and environmental materials. Techniques and Methods 5–D3.. http://pubs.usgs.gov/tm/2007/05D03/pdf/TM5-D3_508.pdf.

[r11] Henn BC, Schnaas L, Ettinger AS, Schwartz J, Lamadrid-Figueroa H, Hernández-Avila M (2012). Associations of early childhood manganese and lead coexposure with neurodevelopment.. Environ Health Perspect.

[r12] Hilson G (2002). The environmental impact of small-scale gold mining in Ghana: identifying problems and possible solutions.. Geograph Jour.

[r13] Lo Y-C, Dooyema CA, Neri A, Durant J, Jefferies T, Medino-Marino A (2012). Childhood lead poisoning associated with gold ore processing: a village-level investigation—Zamfara State, Nigeria, October–November 2010.. Environ Health Perspect.

[r14] Morman SA, Plumlee GS, Smith DB (2009). Application of *in vitro* extraction studies to evaluate element bioaccessibility in soils from a transect across the United States and Canada.. Appl Geochem.

[r15] MSF (Médecins sans Frontières). (2012). Lead Poisoning Crisis in Zamfara State, Northern Nigeria.. http://www.msf.org/article/lead-poisoning-crisis-zamfara-state-northern-nigeria.

[r16] National Institute for Occupational Safety and Health. (2002). Health Effects of Occupational Exposure to Respirable Crystalline Silica. National Institute for Occupational Safety and Health Publication 2002–129.. http://www.cdc.gov/niosh/docs/2002-129/pdfs/2002-129.pdf.

[r17] Plumlee GS, Morman SA (2011). Mine wastes and human health.. Elements.

[r18] UNEP/OCHA (United Nations Environment Programme/Office for the Coordination of Humanitarian Affairs). (2010). Lead Pollution and Poisoning Crisis, Environmental Emergency Response Mission, Zamfara State, Nigeria, September/October 2010.. http://ochaonline.un.org/OchaLinkClick.aspx?link=ocha&docId=1178375.

[r19] U.S. EPA (U.S. Environmental Protection Agency). (2002a). Supplemental Guidance for Developing Soil Screening Levels at Superfund Sites. OSWER 9355.4–24, Appendix A.. http://www.epa.gov/superfund/health/conmedia/soil/pdfs/ssg_main.pdf.

[r20] U.S. EPA (U.S. Environmental Protection Agency). (2002b). Short Sheet: Overview of the IEUBK Model for Lead in Children. OSWER #9285.7–31.

[r21] U.S. EPA (U.S. Environmental Protection Agency). (2007). Method 6200: Field Portable X-Ray Fluorescence Spectrometry for the Determination of Elemental Concentrations in Soil and Sediment.. http://www.epa.gov/osw/hazard/testmethods/sw846/pdfs/6200.pdf.

[r22] U.S. EPA (U.S. Environmental Protection Agency). 2011a Screening Levels for Chemical Contaminants. Available: http://www.epa.gov/region9/superfund/prg/ [accessed 20 November 2012]

[r23] U.S. EPA (U.S. Environmental Protection Agency). (2011b). Exposure Factors Handbook 2011 Edition (Final). EPA/600/R-09/052F.. http://www.epa.gov/ncea/efh/pdfs/efh-complete.pdf.

[r24] von Lindern IH, von Braun MC, Tirima S, Bartrem C. (2011). Zamfara, Nigeria Lead Poisoning Epidemic Emergency Environmental Response, May 2010–March 2011, Final Report to the United Nations Childrens Fund (UNICEF).. http://www.terragraphics.com/Docs/Zamfara_Emergency_Response_UNICEF_Final_Report.pdf.

[r25] WHO (World Health Organization). (2011a). Guidelines for Drinking-water Quality, 4th ed. Geneva:WHO.. http://whqlibdoc.who.int/publications/2011/9789241548151_eng.pdf.

[r26] WHO (World Health Organization). (2011b). Safety Evaluation of Certain Food Additives and Contaminants. Geneva:WHO.. http://whqlibdoc.who.int/publications/2011/9789241660648_eng.pdf.

