# Supplementary material for: Linking Geological and Health Sciences to Assess Childhood Lead Poisoning from Artisanal Gold Mining in Nigeria
Source: Environ Health Perspect. 2013 Mar 22;121(6):744–50. doi: 10.1289/ehp.1206051 (PMC3672918; doi:10.1289/ehp.1206051)
Supplement: (6.3 MB) PDF [file ehp.1206051.s001.pdf]

Environmental Health Perspectives doi:10.1289/ehp.1206051

SUPPLEMENTAL MATERIAL

Linking Geological and Health Sciences to Assess Childhood Lead Poisoning from Artisanal Gold Mining in Nigeria

Geoffrey S. Plumlee[1], James T. Durant[2], Suzette A. Morman[1], Antonio Neri[2], Ruth E. Wolf[1], Carrie A. Dooyema[2,3], Philip L. Hageman[1], Heather A. Lowers[1], Gregory L. Fernette[1], Gregory P. Meeker[1], William M. Benzel[1], Rhonda L. Driscoll[1], Cyrus J. Berry[1], James G. Crock[1], Harland Goldstein[1], Monique Adams[1], Casey L. Bartrem[4,5], Simba Tirima[4,5], Behrooz Behbod[2,3], Ian von Lindern[4], and Mary Jean Brown[2]

[1] U.S. Geological Survey, Denver, Colorado, USA, [2] U.S. Centers for Disease Control and Prevention, Atlanta, Georgia, USA, [3] Epidemic Intelligence Service, U.S. Centers for Disease Control and Prevention, Atlanta, Georgia, USA, [4] TerraGraphics Environmental Engineering, Moscow, Idaho, USA, [5] University of Idaho, Moscow, Idaho, USA

Table of contents

	Page
Supplemental Material, Figure S1.	2
Supplemental Material, Field Sampling Methods	3
Supplemental Material, Table S1	4
Supplemental Material, Table S2	5
Supplemental Material, Figure S2	6
Supplemental Material, Figure S3	7
Supplemental Material, Lead Uptake Calculations	8
Supplemental Material, Figure S4	9
Supplemental Material, References Cited	10

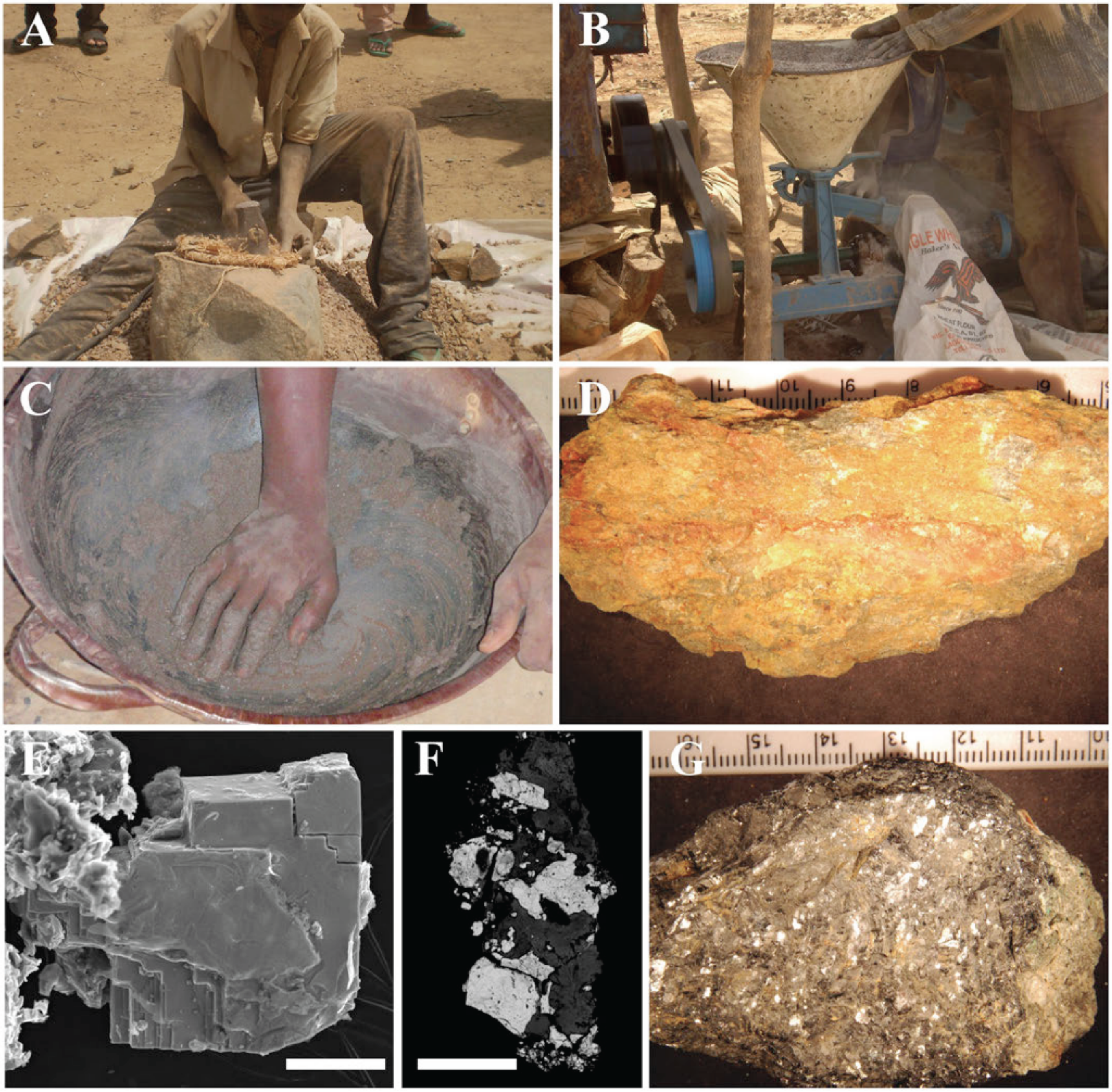

Supplemental Material, Figure S1. A. Ores are initially crushed by hand with sledge hammers, by workers who do not wear any eye or respiratory protection. **B.** Ores are then ground using flour mills installed in central locations in the villages. The grinding generates substantial dust, and the workers do not wear respiratory protection. All workers involved with the crushing and grinding were observed to have extensive deposits of dust and grime on their clothing. **C.** Amalgamation is often done in cooking pots, without the use of gloves or other protection. Photos A–C by Carrie Dooyema and Casey Bartrem. **D.** This "gold" ore sample is a quartz-rich vein ore. The ores have been partially weathered prior to mining, resulting in the orange to red secondary iron oxide minerals visible in the photo. Secondary lead carbonates, oxides and phosphates are abundant in this sample. Scale is in cm. **E.** Small (<0.1 mm) cubic grains of primary lead sulfide (galena) that escaped weathering, such as the one in this Scanning Electron Microscope (SEM) image, can be seen with aid of a hand lens in the sample from photo D. Bar is 25 µm. **F.** Secondary lead carbonates and phosphates from the sample in D are only identifiable using SEM, and appear as brighter phases in this backscatter electron SEM image. Bar is 250 µm. **G.** Sample of "lead" ore with abundant primary galena (shiny gray) and, on the right edge of the sample, a weathering rind of dull gray, white, green and orange secondary minerals. Scale is in cm.

Supplemental Material, Field Sampling Methods

Grab samples of raw ore were obtained from miners at the Dutsen Gomma mine site and in villages. Samples of ore from each of the ore processing steps in Dareta and Yargalma were scooped from storage piles.

Composite soil samples were composed of from 49 to > 100 representative subsamples collected across each area of interest, including: a) village-wide in each village, b) village outskirts (up to 100 m away from village boundaries), and c) areas of known ore processing. Soil subsamples were collected to a consistent depth (2 cm, the depth of non-compacted soil) using the CRREL Multi-Increment Soil Sampling Tool (EnviroStat, Fort Collins, Colorado, USA). Total soil sample weights were >100 grams.

Sweeping was conducted by a resident of the home or family compound at the request of the sampler using the family's broom. Dust from the dirt floor of the room or family compound sub-area was swept into a central pile and a minimum 5 aliquot composite was collected using a pre-cleaned stainless steel spoon. Sweeping samples weights ranged from 10-100 grams. See additional details in Dooyema et al. (2012).

Plant foodstuff samples were collected both raw from storage bins and following home or market processing (e,g., grinding by mortar/pestle or flourmill) and food preparation.

Ore, soil, sweep, and plant foodstuff samples were collected into resealable plastic bags.

Supplemental Material, Table S1. Laboratory sample preparation and analysis methods. Sample splits for different analyses were obtained by standard cone and quartering methods (Taggart, 2002). Appropriate QA/QC analyses of standard reference materials, duplicate sample splits, analytical duplicates, and blanks were performed for all methods.

**Analysis type and sample type analyzed**	**Analytical method**	**Sample preparation and method details**	**References**
Spot chemical analyses of heterogeneous raw ore samples	*Handheld X-Ray Fluorescence in laboratory* (Thermo Niton XLt 950, Billerica, MA, USA)	No sample preparation needed. Lead bearing standard reference materials (NIST soils 2710a, 2711) were used to verify calibration	EPA (2007)
Quantitative particle size distribution of processed ores, soils, sweep samples	*Laser diffraction analysis of sample by settling through aqueous suspension*	Sample sieved to < 2 mm using stainless steel sieve.	Ryżak and Bieganowski (2011)
Mineralogy (qualitative abundance) of processed ores, soils, sweep samples	*Powder X-ray Diffraction*	Sample sieved to < 2 mm using stainless steel sieve, ground to < 100 µm and homogenized using agate mortar and pestle.	Jenkins and Snyder (1996)
Mineralogy, size, shape, and chemistry of individual particles in solids, plant samples	*Scanning Electron Microscopy*	Samples analyzed included 1) particles adhered to an SEM mount via double-sided tape, and 2) polished epoxy grain mounts of representative particles.	Meeker et al. (2005)
Total chemical composition of processed ores, soils, sweep samples	*Inductively Coupled Plasma-Mass Spectrometry (ICP-MS)* for 42-element suite	Sample sieved to < 2 mm using stainless steel sieve, ground to < 100 µm and homogenized using agate mortar and (hydrochloric, nitric, perchloric, hydrofluoric).	Briggs and Meier (2002)
	Continuous flow-cold Vapor-Atomic Fluorescence Spectroscopy (CVAFS) for total mercury	Sample sieved to < 2 mm using stainless steel sieve, ground to < 100 µm and homogenized using agate mortar and pestle, digested using nitric acid and sodium dichromate.	Hageman (2007a)
Deionized water leach of processed ores, soils, sweep samples	*USGS Field Leach Test*. Leachate analyzed by: ICP-MS for 44 element suite; ion chromatography for sulfate, fluoride, nitrate, and chloride; CVAFS for mercury	Sample sieved to < 2 mm using stainless steel sieve. 5 g solid added to 100 ml deionized water. Mixture agitated by hand for 5 minutes. Leachate filtered to < 0.45 µm using nitrocellulose syringe filter prior to analysis.	Hageman (2007b); Lamothe et al. (2002)
Gastric bioaccessibility of elemental toxicants from processed ores, soils, sweep samples	*In-Vitro Bioaccessibility Assessment (IVBA)*. Simulated gastric fluid leachate analyzed by: ICP-MS for 44 element suite; CVAFS for mercury.	Sample sieved to < 250 µm using stainless steel sieve. 1 g solid added to 100 ml simulated gastric fluid (pH 1.5, hydrochloric acid in deionized water, 30.3 g/L glycine). Mixture agitated at 37°C for 1 hour. Leachate filtered to < 0.45 µm using nitrocellulose syringe filter prior to analysis.	Drexler and Brattin (2007); Morman et al. (2009); Lamothe et al. (2002)
Total chemical composition of processed and unprocessed grain, spice and herb foodstuff samples	*Inductively Coupled Plasma-Atomic Emission Spectrometry (ICP-AES)* for a 40-element suite.	Plant materials homogenized using food processor then digested using nitric acid and hydrogen peroxide for 45 minutes each in a microwave digestion system (Multiwave 3000, Anton Paar, Grasz, Austria).	Wolf et al. (2009); Briggs (2002)
	Continuous flow-cold Vapor-Atomic Fluorescence Spectroscopy (CVAFS) for total mercury.		Wolf et al. (2009); Hageman (2007a)

Supplemental Material, Table S2. Qualitative summary of ore-related minerals found in unprocessed and processed "gold" and "lead" ore samples, soil samples, and eating area sweep samples collected by CDC/TG from Dareta, Yargalma, and the Dutsen Gomma mine. Formal mineral names (when identified with certainty) are listed in parentheses. A blank entry does not mean that the mineral is not present, only that it has not been identified. The results are from hand sample observation, powder X-ray diffraction analysis, and SEM analysis. Major: Greater than 40 % by weight. Moderate: 20-40 % by weight. Minor: 2-20% by weight. Trace: < 2% by weight.

	"Gold" ores	"Lead" ores	Soils	Sweep samples
Primary Vein Minerals				
Crystalline silica (quartz)	Major	Moderate	Moderate	Moderate
Lead sulfide (galena)	Minor	Major	Trace	Trace
Zinc sulfide (sphalerite)	Trace	Minor		
Iron sulfide (pyrite)	Trace	Minor	Trace	Trace
Copper iron sulfide (chalcopyrite)		Trace		
Iron arsenic sulfide (arsenopyrite)	Trace	Trace		
Secondary Vein Minerals (formed by weathering of primary ores)				
Lead carbonate (cerussite)	Moderate	Minor	Moderate	Moderate
Lead oxide (massicot)	Moderate	Minor	Moderate	Moderate
Lead phosphates (pyromorphite, plumbogummite)	Minor	Minor	Minor	Minor
Lead sulfate (anglesite)	Minor	Major	Minor	Minor
Lead vanadate			Trace	Trace
Lead-copper-potassium-aluminum sulfate (beaverite)	Minor		Trace	Trace
Lead-bearing iron oxides	Moderate		Moderate	Moderate
Lead-bearing manganese oxides	Minor		Minor	Minor
Lead-rich tungstate	Trace			
Copper sulfate	Trace	Trace		
Arsenic oxide			Trace	
Copper sulfide (covellite)		Trace		

A

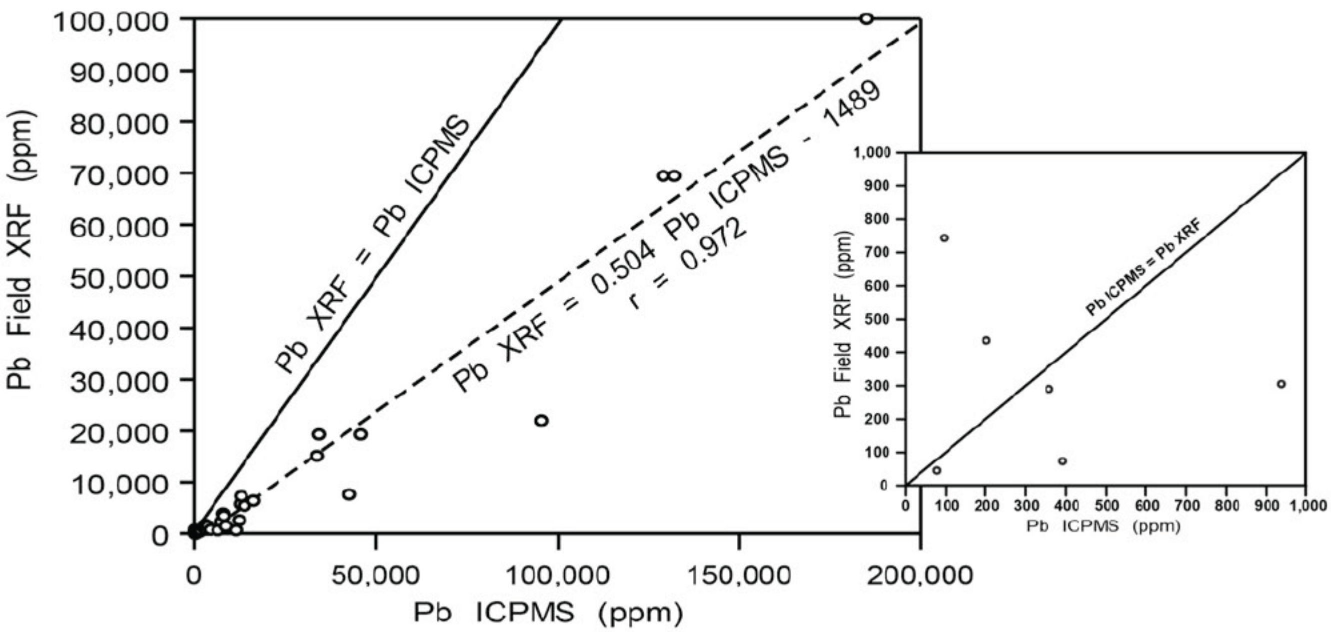

B

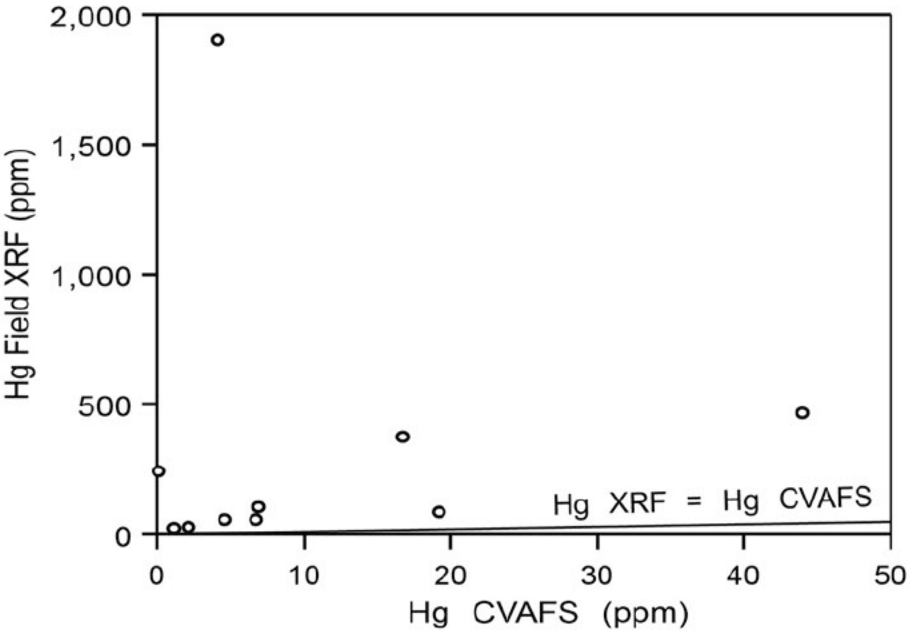

Supplemental Material Figure S2. A. Large plot shows total lead concentrations measured by in the field by CDC and TG field crews for soil and sweep samples using handheld XRF (Y axis) compared to those measured in the laboratory using ICP-MS (X axis) on the same samples sieved to <2mm and ground, then digested using four acids. Small inset plot shows details for samples with concentrations measured below 1,000 ppm. **B.** Plot showing total mercury concentrations measured in soil and sweep samples in the field using handheld XRF (Y axis) compared to those measured in the laboratory using CVAFS (X axis) on the same samples sieved to <2mm, ground, then digested with nitric acid and hydrogen peroxide.

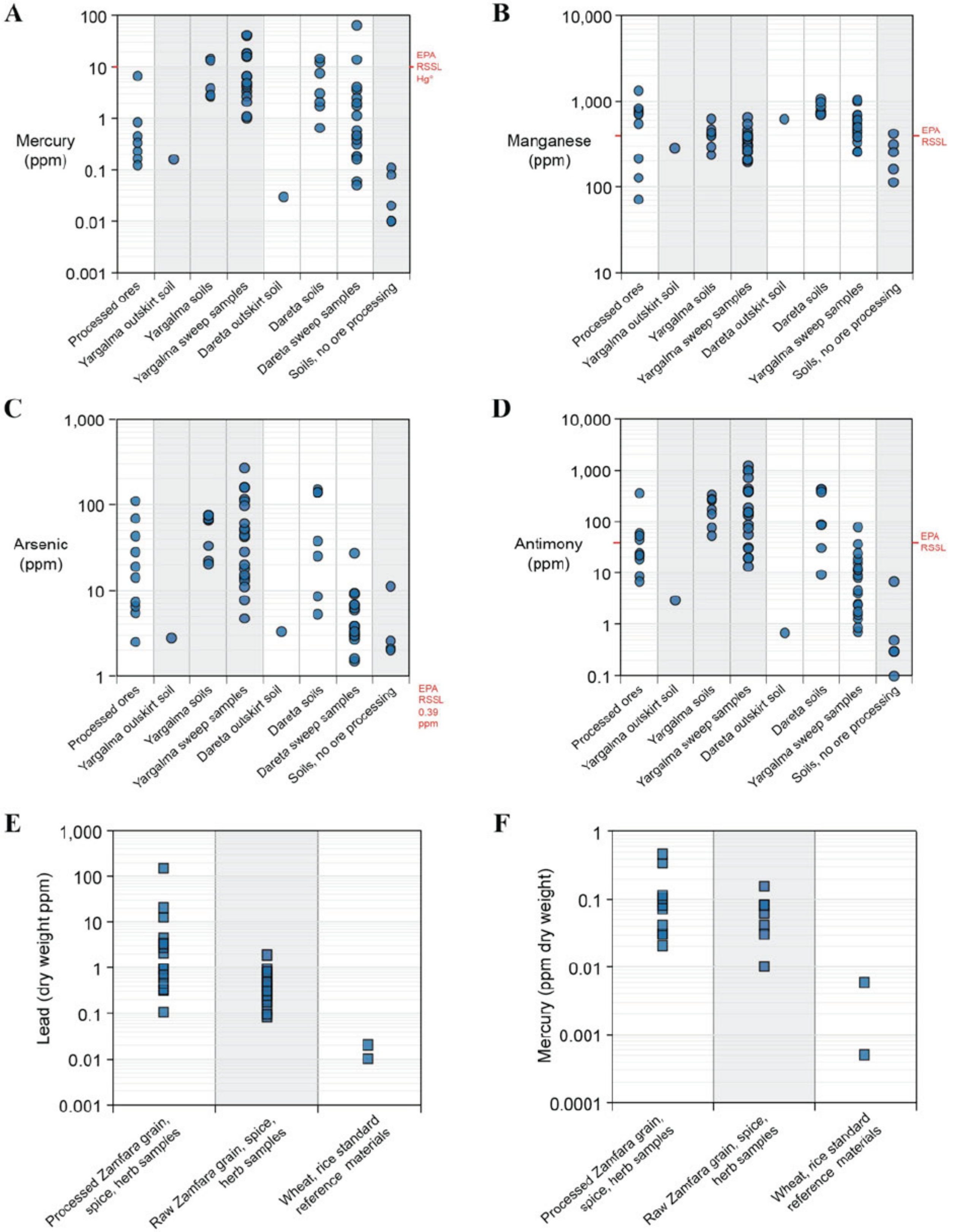

Supplemental Material, Figure S3. A–D: Plots showing the total chemical concentrations of mercury by CVAFS and manganese, arsenic, and antimony by ICP-MS in processed ores, village outskirt soils, village soils, and eating area sweep samples from Dareta, Yargalma, and composite soil samples from five villages where no ore processing occurred. US EPA Residential Soil Screening Levels (RSSLs) are shown as red dashes for comparison. **E, F.** Plots showing total lead and mercury concentrations in processed and unprocessed grain, spice, and herb foodstuff samples from Zamfara compared to NIST wheat and rice standard reference materials.

Supplemental Material, Lead Uptake Calculations

Lead uptake levels were calculated using soil consumption rates, our bioaccessibility results (journal article, Fig. 3), and the method described by Drexler and Brattin (2007) to convert bioaccessible lead into bioavailable lead for uptake modeling.

The gastric-bioaccessible lead concentration we measured for each sample was first recalculated into ppm mass basis:

$$[\text{gastric bioaccessible Pb}]\ (\text{ppm mass basis, mg Pb leached/kg solid}) =$$

$$[\text{mg Pb/kg leachate}] \cdot [100 \text{ kg leachate/1 kg solid}]$$

This gastric-bioaccessible lead concentration was then recalculated into bioavailable lead concentration (mg bioavailable Pb/kg solid) using the formulas of Drexler and Brattin (2007):

$$[\text{Bioavailable Pb}] = 0.5 \cdot ((0.878 \cdot [\text{Gastric bioaccessible Pb}]) - 0.028)$$

Estimated daily lead uptake (µg Pb/day) was then calculated using published daily soil consumption rates (mg/day) and appropriate mass conversion factors:

$$\text{Daily Pb uptake} = [\text{Bioavailable Pb}] \cdot \text{Daily soil consumption} \cdot (1/1000)$$

We used published soil consumption rates (e.g., US EPA 2011) of 10 and 500 mg/day, a range for children under clean (unlikely for the villages) to extremely dusty (more plausible) conditions. Results of uptake calculations are plotted in Figure 4 of the journal article.

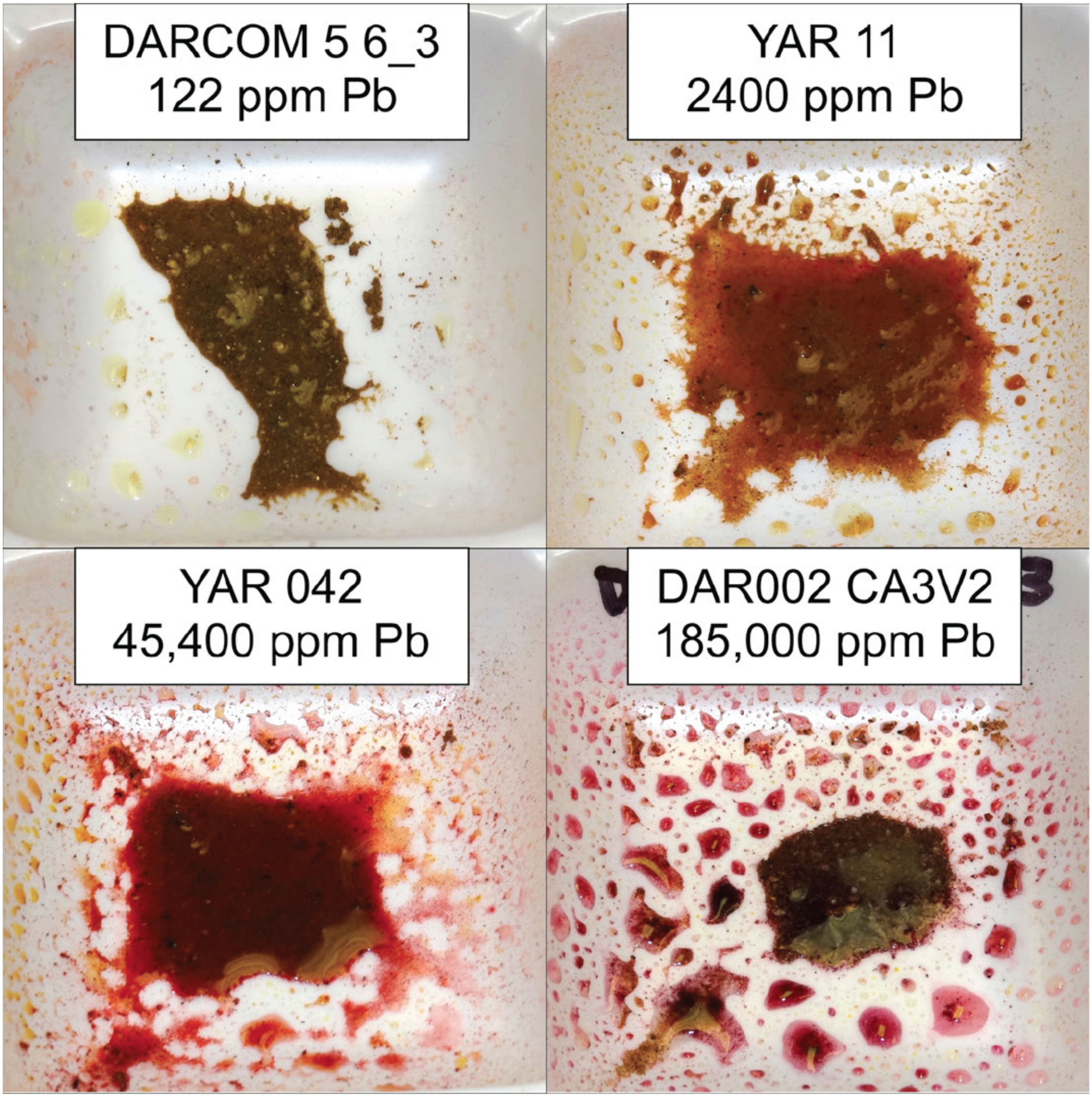

Supplemental Material, Figure S4. These photos show the successive changes in color associated with increasing lead content of four Zamfara soil samples as indicated by the sodium rhodizonate chemical indicator used in NIOSH Method 9105 for chemical detection of lead (Esswein and Ashley 2003). For each photo caption, the top line is the sample ID and the bottom line is the total lead concentration as measured using ICP-MS on the sample. As per the method instructions for analyzing surface wipes, each sample was first sprayed three times with acetic acid, then three times with the rhodizonate solution. These results suggest that the test might be a feasible way for miners to detect lead-rich gold ores, when no other methods such as handheld XRF are available. However, there are funding and logistical challenges associated with getting large numbers of these test kits to Zamfara mine sites. Another logistical challenge to application of this method in the field in Zamfara is that rhodizonate solution is unstable in the heat and must be refrigerated once made.

Supplemental Material, References Cited

Briggs PH. 2003. The determination of forty elements in geological and botanical samples by inductively coupled plasma-atomic emission spectrometry. US Geol Survey Open File Rept 02-223-G.

Briggs PH, Meier AL. 2002. The determination of forty-two elements in geological materials by inductively coupled plasma-mass spectrometry. US Geol Survey Open File Rept 02-223-I.

Dooyema C, Neri A, Lo Y-C, Durant J, Dargan PI, Swarthout T, et al. 2012. Outbreak of fatal childhood lead poisoning related to artisanal gold mining in northwestern Nigeria, 2010. Environ Health Perspect 120:601–607.

Drexler JW, Brattin WJ. 2007. An in vitro procedure for estimation of lead relative bioavailability, with validation. Human Ecol Risk Assess 13:383–401.Hageman PL. 2007a. Determination of mercury in aqueous and geologic materials by Continuous Flow-Cold Vapor-Atomic Fluorescence Spectrometry (CVAFS). US Geol Surv Techs Methods Rept 5—D2.

Esswein EJ, Ashley K. 2003. Lead in Dust Wipes by Chemical Spot Test, NIOSH Method 9105. NIOSH Manual of Analytical Methods, 4th ed., 3rd. Supplementary Material (2003). Available: www.cdc.gov/niosh/docs/2003-154/pdfs/9105.pdf.

Hageman PL. 2007a. Determination of mercury in aqueous and geologic materials by Continuous Flow-Cold Vapor-Atomic Fluorescence Spectrometry (CVAFS). US Geol Surv Techs Methods Rept 5—D2.

Hageman PL. 2007b. U.S. Geological Survey field leach test for assessing water reactivity and leaching potential of mine wastes, soils, and other geologic and environmental materials. US Geol Surv Techniques Methods Rept 5—D3.

Jenkins R, Snyder RL. 1996 Introduction to X-ray Powder Diffractometry. Chemical Analysis v. 138 (JD Winefordner, ed.), Wiley-Interscience Publication, New York.

Lamothe PJ, Meier AL, Wilson SA. 2002. The determination of forty four elements in aqueous samples by inductively coupled plasma-mass spectrometry. US Geol Survey Open File Rept 02-223-H.

Meeker GP, Sutley SJ, Brownfield IK, Lowers HA, Bern AM, Swayze GA, Hoefen TM, Plumlee GS, Clark RN, Gent CA. 2005. Materials characterization of dusts generated by the collapse of the World Trade Center. In *Urban Aerosols and Their Impacts: Lessons Learned from the World Trade Center Tragedy* (JS Gaffney, NA Marley, eds.), American Chemical Society Series 919: 84-102.

Morman SA, Plumlee GS, Smith DB. 2009. Application of *in vitro* extraction studies to evaluate element bioaccessibility in soils from a transect across the United States and Canada. Appl Geochem 24: 1454-1463.

Ryżak M, Bieganowski, A. 2011. Methodological aspects of determining soil particle-size distribution using the laser diffraction method. J Plant Nutr Soil Sci. 174:624–633.

Taggart JE Jr (ed.). 2002. Analytical methods for chemical analysis of geologic and other materials. US Geol Surv Open-File Rept 02–0223.

US EPA. 2007. SW-846 On-line. Test Methods for Evaluating Solid Wastes Physical/Chemical Methods. Available: http://www. epa.gov/osw/hazard/testmethods/sw846/pdfs/6200.pdf [accessed 24 February 2012].

US EPA. 2011. Exposure factors handbook 2011 Edition (Final). EPA/600/R-09/052F.

Wolf RE, Todd AS, Brinkman S, Lamothe PJ, Smith KS, Ranville JF. 2009. Measurement of total Zn and Zn isotope ratios by quadrupole ICP-MS for evaluation of Zn uptake in gills of brown trout (*Salmo trutta*) and rainbow trout (*Oncorhynchus mykiss*). Talanta 80: 676–684.
